# Recording the Centric Relation in an Edentulous Patient Using the Existing Complete Denture Mounted with Gothic Arch Tracer

**DOI:** 10.1155/2021/7819818

**Published:** 2021-10-18

**Authors:** Xu Wei, X. F. Meng, Lang Guo, Xiaoping Luo, Wei Han

**Affiliations:** ^1^Department of Prosthodontics, Nanjing Stomatological Hospital, Medical School of Nanjing University, Nanjing, China; ^2^Department of Otolaryngology, Nanjing Stomatological Hospital, Medical School of Nanjing University, Nanjing, China

## Abstract

An 80-year-old man sought treatment at our hospital. He was dissatisfied with his old complete denture due to its poor stability and retention. The old complete denture had been used for about 20 years. The prolonged use of an unsuitable complete denture led the patient to be accustomed to unilateral mastication (UM). Due to the patient long-term habitual mandibular deviation, using the physiological technique to get the centric relation (CR) achieved an incorrect horizontal maxillomandibular record. This clinical report presents a technique using the existing complete denture mounted with a Gothic arch tracer to determine the CR. This technique is an inexpensive, simple, and reliable method that allows fabricating the final impression and obtaining the maxillomandibular relationship record (MMRR) in one step.

## 1. Introduction

The determination of the correct MMRR is one of the most sensitive and rigorous procedures in complete denture treatment. For some patients, it is difficult to determine the CR due to the habitual mandibular deviation induced by previous inadequate dentures. An inexperienced dentist is prone to obtain an erroneous MMRR in this situation. The Gothic arch tracer has been acknowledged as one of the most reliable and accurate patient-guided methods for obtaining the CR [1]. A method for fabricating the final impression and determining the CR using the existing complete denture mounted with a Gothic arch tracer in one step is described in the present article.

## 2. Case Report

**Figure 1:**
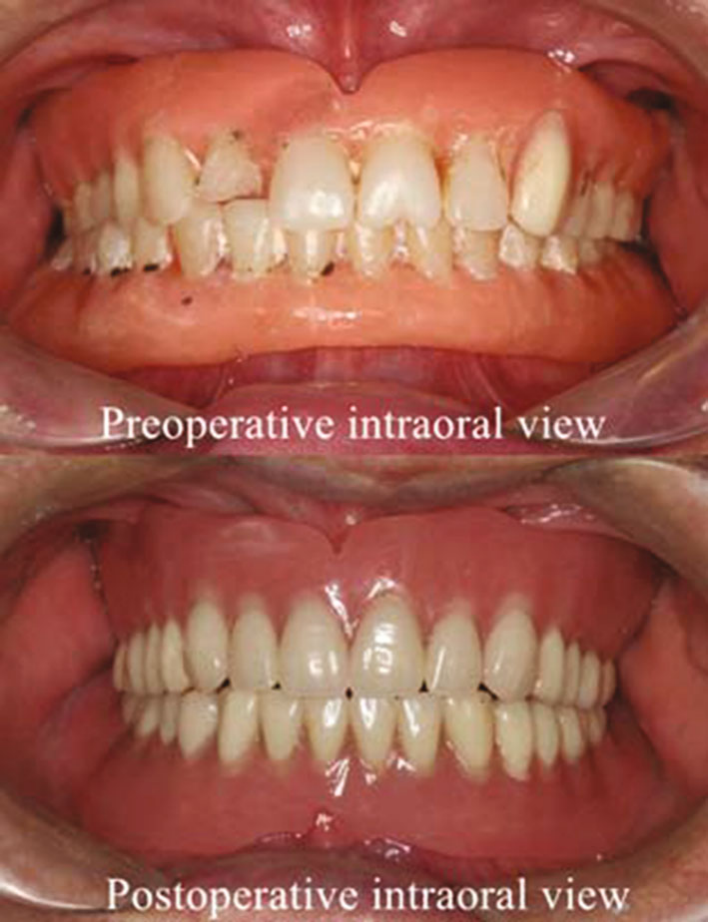
Intraoral view of the old complete denture and the new complete denture.

An 80-year-old man came to our hospital for a new complete denture. His complete denture had been used for about 20 years. He was dissatisfied with the complete denture due to its poor retention, frequent ulcers, and unaesthetic appearance. The old complete denture showed a decreased vertical dimension of occlusion. The extraoral examination revealed the patient's mandible deviated to the right of the old complete denture (Figure 1).

**Figure 2:**
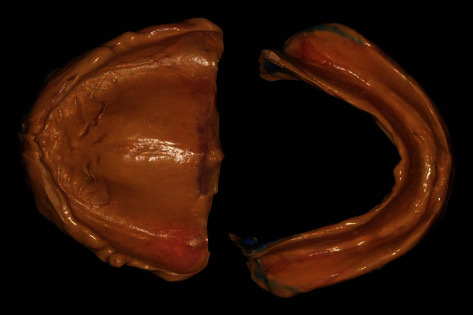
The final function impression with the existing complete denture.

The worn occlusal surface of the maxillary artificial teeth was covered with the wax rim, checking the parallelism of the maxillary horizontal plane with the interpupillary line in the frontal view and the parallelism of the occlusal template with the camper line (nasoauricular plane) in the lateral view. The occlusal vertical dimension (OVD) was determined using the interocclusal rest space method. Softened wax rim was added onto the occlusal surface of the mandibular denture. The patient was asked to swallow once the predetermined OVD had been reached. Checking the existing complete denture bases' extension, the denture bases should be ground away approximately 2 millimeters above the deepest part of the muccobuccal fold. Then placing silicone border molding material (Virtual Heavy, Ivoclar Vivadent AG, Liechtenstein) to the existing maxillary complete denture for border molding, the patient was asked to make three functional movements: saying “woo,” saying “eee,” and sucking the operator's finger. Using a brush to distribute the high-flow silicone impression material (Virtual Light, Ivoclar Vivadent AG, Liechtenstein) evenly onto the tissue surface of the maxillary existing complete denture and inserting the denture into the mouth, the patient was asked to bite gently and repeat making the three functional movements. The mandibular final impression was taken in the same manner. Besides the three maxillary functional movements, the patient should be asked to move the tongue from side to side, push the back of the denture with the tongue, and swallow in a closed-mouth position during manufacturing the mandibular final impression. The final functional impression was obtained using the closed-mouth impression technique (Figure 2).

**Figure 3:**
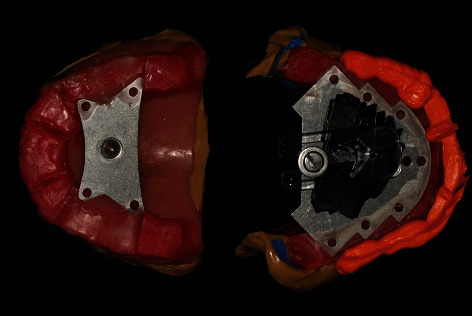
Mounting the Gothic arch tracer to the existing complete denture and the Gothic arch apex of the CR was obtained.

The Gothic arch tracer (CRS 10, Candulor Registration Set, Ivoclar Vivadent AG, Liechtenstein) was mounted on the existing complete denture. The tracing stylus was set on the maxilla, the tracing recording table was set on the mandible, and the recording table was parallel to the camper's line. Repetitious training was performed for the patient's mandibular protrusion and laterotrusion movements until an accurate arrow point tracing (Gothic arch apex, GoA) was obtained. A fine hole in the plastic cover was placed at the GoA point to engage the stylus in the CR when the interocclusal recording material (O-bite, DMG, Germany) was being made (Figure 3).

**Figure 4:**
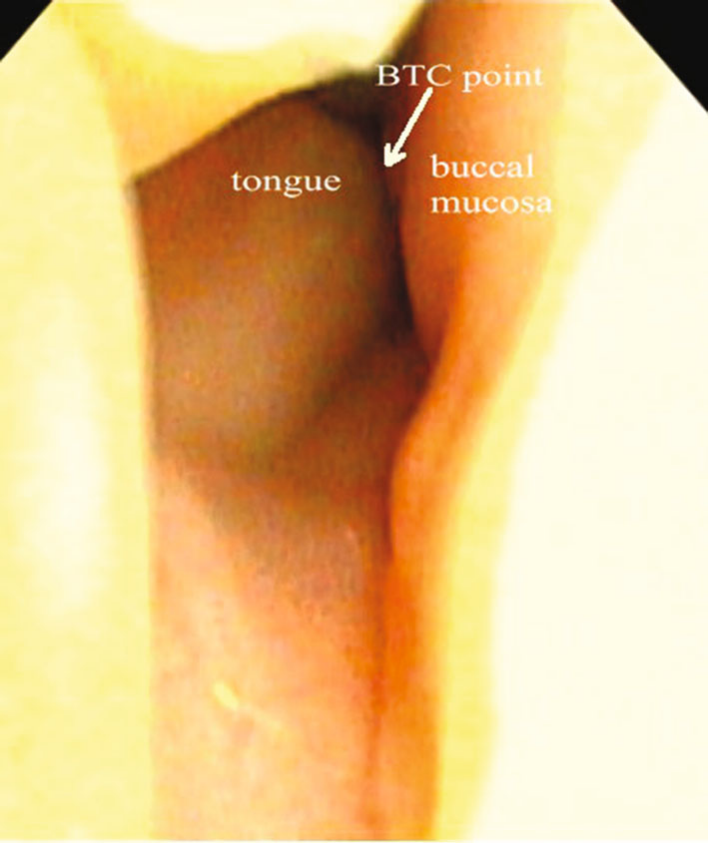
Electrolaryngoscope observation of the BTC point during mouth closure.

The working models were mounted in an articulator (Amann Girbach, Vorarlberg, Austria) with a facebow transfer (Amann Girbach, Vorarlberg, Austria). To confirm the jaw position and aesthetics, wax denture try-in was done and the patient was satisfied with his appearance. Fortunately, the maxillomandibular relation remained stable in maximal intercuspal position. Harmonious occlusion and articulation were achieved. If the patient repeatedly goes into an accustomed position, the patient was treated with a therapeutic complete denture, of which the maxillary premolars and molars were artificial ceramic teeth and mandibular premolar and molar region was just like a splint. A therapeutic complete denture was used to rectify maxillomandibular relation until a stable jaw position is achieved. In a customary manner, a new complete denture was finished. The retention, stability, and occlusion of the complete denture were good. According to the sealing mechanism of a mandibular suction-effective denture [2], we used an electrolaryngoscope to observe the posterior border seal on the tongue and buccal mucosa above the polished surface in the retromolar pad region; the buccal mucosa and tongue side contact point (BTC point) could be seen during mouth closure (Figure 4) [2]. Recall appointments were scheduled 1 month, 3 months, and 6 months; the patient reported improvements in chewing and speaking in addition to his appearance (Figure 1).

## 3. Discussion

The accuracy of MMRR is essential for an appropriate complete denture [3]. Fenlon et al. has demonstrated a positive correlation between complete denture usage and the accuracy of the MMRR [4]. The Gothic arch tracer is used to obtain the CR for a new complete denture with record base and wax rim [5]. Due to its technique sensitivity of the procedure, the Gothic arch tracer remains a largely underutilized tool. The accurate Gothic arch tracing needs stable and retentive bases to obtain the correct MMRR. Using the existing complete denture can skip the procedure of making the temporal record base, and the stability and fitness of the existing complete denture bases with final impressions are better than the temporal record base or wax base. The Biofunctional Prosthetic System (BPS, Ivoclar Vivadent AG, Liechtenstein) for complete dentures uses the “Gnathometer,” which consists of custom trays and the Gothic arch tracer, to determine the CR and fabricate the final impression in one step. However, the “Gnathometer” is an expensive and special device. Using the existing complete denture mounted with Gothic arch tracer is an inexpensive, simple, and reliable alternative that can make impression taking and bite registration in one step.

## 4. Conclusions

Recording the centric relation of edentulous patients using the existing complete denture mounted with Gothic arch tracer is an inexpensive, simple, and reliable method. It can fabricate the final impression and obtain the maxillomandibular relationship record (MMRR) in one step.
